# Combining Neuroprotectants in a Model of Retinal Degeneration: No Additive Benefit

**DOI:** 10.1371/journal.pone.0100389

**Published:** 2014-06-23

**Authors:** Fabiana Di Marco, Mattia Di Paolo, Stefania Romeo, Linda Colecchi, Lavinia Fiorani, Sharon Spana, Jonathan Stone, Silvia Bisti

**Affiliations:** 1 Department of Biotechnology and Applied Clinical Science, University of L'Aquila, L'Aquila, Italy; 2 Discipline of Physiology and Bosch Institute, University of Sydney, Sydney, New South Wales, Australia; 3 ARC Centre of Excellence in Vision Science, The Australian National University, Canberra, Australia; Dalhousie University, Canada

## Abstract

The central nervous system undergoing degeneration can be stabilized, and in some models can be restored to function, by neuroprotective treatments. Photobiomodulation (PBM) and dietary saffron are distinctive as neuroprotectants in that they upregulate protective mechanisms, without causing measurable tissue damage. This study reports a first attempt to combine the actions of PBM and saffron. Our working hypothesis was that the actions of PBM and saffron in protecting retinal photoreceptors, in a rat light damage model, would be additive. Results confirmed the neuroprotective potential of each used separately, but gave no evidence that their effects are additive. Detailed analysis suggests that there is actually a negative interaction between PBM and saffron when given simultaneously, with a consequent reduction of the neuroprotection. Specific testing will be required to understand the mechanisms involved and to establish whether there is clinical potential in combining neuroprotectants, to improve the quality of life of people affected by retinal pathology, such as age-related macular degeneration, the major cause of blindness and visual impairment in older adults.

## Introduction

The central nervous system in mammals has only a limited ability to repair its neuronal circuitry. Its functional stability is achieved by ensuring the stability of individual neurons and by redundancy that enables normal function despite substantial loss of neurons. Age-related loss of retinal stability results in diseases such as age related macular degeneration (AMD).

Inflammation is an important feature of the aged retina and in many retinal diseases, including AMD. Recent studies have demonstrated that exposure to 670 nm light reduces inflammation in the retina undergoing degeneration [1], [2], [3], mitigates the light-induced upregulation of Müller cell- specific markers, for example glial fibrillary acid protein (GFAP) [5] and vimentin [1], [3], and reduces lipid peroxidation and complement activation in degenerating retina [2], [3].

Saffron has been used for a long time in traditional medicine. Its effectiveness as a neuroprotectant was pioneered by Maccarone and colleagues [4], who showed that dietary saffron maintains photoreceptor morphology and function after exposure to damaging light in rat retina, and reduces the overexpression of fibroblast growth factor 2 (FGF-2). This neuroprotective action of saffron has been confirmed in models of photoreceptor degeneration [5] and Parkinson's disease [6] and in clinical trials with AMD [7], [8].

Microarray analysis [9] showed that both PBM and saffron treatment were able to change the gene expression induced by light damage, but their effects were not identical. Preconditioning by both PBM and saffron mitigated a damage-induced reduction in *GPX3*, which codes for a glutathione peroxidase; and reduced the expression of *CCL2*, which codes for a cytokine which recruits monocytes, memory T-cells and dendritic cells to sites of inflammation; and both reduced the expression of many ncRNAs. Saffron preconditioning, but not PBM, on the other hand, regulated *EDN2*, which codes for a vasoconstrictive peptide, and the ncRNAs regulated by saffron and PBM differed significantly (Tables 4 and 5 in [9]), These differences suggested that the simultaneous application of the two neuroprotectants might have an additive and more powerful protective activity.

Our previous study has described the time course of protection for dietary saffron and photobiomodulation (PBM), in an animal model of light damage [5]. Both treatments are effective in reducing retinal degeneration, and present low toxicity. This paper describes a first attempt to define their protective efficacy when simultaneously applied.

## Methods

### Light damage model

All experiments conducted were in accordance with the policies of the Association for Research in Vision and Ophthalmology (ARVO) and with the approval of the Animal Ethics Committee at the University of Sydney (Approval number: K22/5-2009/2/5003). Animals were raised and experiments conducted in cyclic 5 lux light (12 hrs: 12 hrs). Adult Sprague Dawley (SD) albino rats were born and raised in dim cyclic light conditions (12 h at 5 lux, 12 h dark)

Light damage (LD) was generated by exposing the animals to 1000 lux light for 24 h. The light was generated by fluorescent tubes located above the cage. For the exposure period, the animals were provided with food and water from containers on the floor of the cage, to ensure consistent exposure to the light. After LD the animals were returned to dim cyclic illumination for a post-exposure period of one week (1 w). The animals were euthanized (Lethobarb 60 mg/kg intraperitoneal) and retinal tissues were obtained for analysis.

Five groups of animals were used:

- Control: These animals (n = 4) were raised in 5 lux cyclic light, as above.
- Light damaged (LD) control: These animals (n = 10) were raised in dim cyclic light, then exposed to bright light for 24 h, and returned to dim cyclic light for 1 w.
- Saffron-conditioned LD: These animals (n = 10) were raised in dim cyclic light and, prior to exposure to bright light, were preconditioned for 10 days with saffron at 1 mg/kg/day. Saffron (stigmata of *Crocus sativus*, “L'Aquila Saffron”, Italy) was soaked in water (at 2 mg of spice/ml H_2_O) and 12 h was allowed for the major antioxidants, which are water soluble [10], to dissolve. The solute was then fed to the rats by injecting a small volume into a piece of the vegetable matrix, which the animal readily ingested. The volume for each daily feed was calculated to provide the solutes from 1 mg of saffron/kg body weight.
- Photobiomodulation (PBM) conditioned LD: These animals (n = 10) were raised in dim cyclic light, exposed to bright light and kept for a further week, as above. For 7d prior to exposure to the bright light, each animal was exposed to 670 nm red light from a WARP 75 source (Quantum Devices Inc, Barneveld, WI, USA). Animals were gently restrained under a plexiglass platform with the eyes ∼2.5 cm below the platform. The WARP 75 device was placed on top of the platform and turned on for 3 min. This arrangement provided a fluence of 4.0–4.5 J/cm^2^ at the eye, calculated from an estimate of power at 2.5 cm from the LED array, made using a calibrated sensor provided by Quantum Devices (Barneveld, Wisconsin). The animals did not hide from or appear agitated by the red light.
- Combined conditioned: These animals (n = 10) were raised in dim cyclic light, exposed to bright light and kept for a further week, as above. For 10d prior to the exposure to bright light, each was exposed simultaneously to both the saffron and the PBM conditioning described above.

### Preparation of retinal material

The superior aspect of the eye was marked with an indelible marker by a stitch in the conjunctiva, after anaesthesia and prior to euthanasia. After euthenasia, the eyes were dissected free and fixed by immersion in 4% paraformaldehyde fixative buffer at 4°C for 1 h. After three rinses in 0.1 M phosphate-buffered saline (PBS), eyes were left overnight in a 15% sucrose solution to provide cryoprotection. Eyes were embedded in mounting medium (Tissue Tek OCT compound; Sakura Finetek, Torrance, CA) by snap freezing in liquid nitrogen. Cryosections were cut at 20 µm (CM1850 Cryostat; Leica, Wetzlar, Germany) with the eyes oriented so that the sections extended from superior to inferior edge. Sections were mounted on gelatin and poly-L-lysine-coated slides and were then dried overnight in 50°C oven and stored at −20°C until processed.

### Detection of cell death (TUNEL)

Sections were labelled for apoptotic cell death using the terminal deoxynucleotidyl transferase dUTP nick end labelling (TUNEL) technique [11] following protocols published previously [12]. To demonstrate cellular layers, sections were also labelled with the DNA-specific dye bisbenzimide (Calbiochem, La Jolla, CA), by incubating them for 2 min in a 1∶10.000 solution in 0.1 M PBS. Sections cut adjacent to or through the optic nerve head were chosen, to minimise variations in retinal length and position. Counts of TUNEL+ profiles (apoptotic cells) were made using a calibrated 20 x objective and an eyepiece graticule. Each section was scanned from the superior to inferior edge, and the number of TUNEL+ profiles was recorded for each 400 µm length of the section. Counts were averaged from at least four sections per animal and were recorded separately for the outer nuclear layer (ONL) and inner nuclear layer (INL).

### Immunohistochemistry: GFAP staining

Retinal sections were washed with 0.1 M PBS (10 min twice) and incubated in 10% normal goat serum in 0.1 M PBS for 1 hour at room temperature, to block non-specific binding. Sections were then incubated overnight at 4°C in rabbit polyclonal anti GFAP (1∶700; DakoCytomation, Campbellfield, Australia). After 3 rinses in PBS for 10 minutes each, sections were incubated with an appropriate secondary antibody (1∶1.000 ALEXA Fluor 594; Molecular Probes, Invitrogen Carlsbad, CA), for 1 h at room tempertaure

### Outcome measures

Three measures of neuroprotection were used, the surviving population of photoreceptors, the rate of photoreceptor death, and the expression of the stress-inducible protein GFAP in Müller cells. All were assessed 1 w after exposure to damaging light.

#### Photoreceptor survival

We estimated photoreceptor survival by measuring the thickness of the outer nuclear layer (ONL). Specifically, we recorded the ratio of the thickness of the ONL to the thickness of the retina (from the inner to the outer limiting membrane), measured at 0.40 mm intervals, from the superior to the inferior edge of the retina. The ratio of ONL to retinal thickness was used as a measure of ONL thickness, rather than the absolute thickness of the ONL (µm), to compensate for oblique sectioning.

#### Extent of GFAP labelling

We measured the length of Müller cells along which GFAP expression was evident (µm), as a proportion of retinal thickness (from the inner to the outer limiting membrane), This was recorded at 0.4 mm steps along retinal sections, from the superior to the inferior edge. Measurements were made in at least 2 sections from one eye of each animal studied.

### Electroretinographic Recording

Electroretinograms (ERGs) were recorded in control and treated animals 1 day before and 1 week after high intensity light exposure (light damage LD). Albino rats were previously dark adaptated overnight. Ketamine : xylazine anaesthesia was used with intra peritoneal injection of 100 mg/kg ketamine, 12 mg/kg xylazine (Ketavet 100 mg/ml, Intervet production srl; Xylazine 1 g, Sigma Co.). Corneas were anesthetized with a drop of novocaine, and pupils were dilated with 1% atropine sulfate (Allergan, Westport, IR). Body temperature was maintained at 37±0.5°C with a heating pad controlled by a rectal temperature probe. Recordings were made from the left eyes, with a gold electrode loop (2 mm in diameter) placed on the cornea while the right eye was fully covered with a bandage. The reference electrode was placed on the right cornea under the bandage, and the ground electrode was inserted in the anterior scalp, between the eyes. The rat's head was positioned just inside the opening of the Ganzfeld dome (Biomedica Mangoni, Pisa, Italy). This electronic flash unit generated flashes of a range of intensities from 0.001–100 cd/m^2^. Responses were recorded over 300 ms plus 25 ms of pre-trial baseline, amplified differentially, bandpass filtered at 0.3 to 300 Hz, digitized at 0.25- to 0.3-ms intervals by a personal computer interface (LabVIEW 8.2; National Instruments, Milan, Italy), and stored on a disc for processing. Responses from several trials were averaged (*n* = 5), with an interstimulus interval ranging from 60 seconds for dim lights to 5 minutes for the brightest flashes. The amplitude of the b-wave was measured from the most negative point of the average trace to the highest positive point, without subtracting oscillatory potentials. The distributions of ERG response, across several experimental groups at different light intensities, were described by means and standard deviations. The between-group differences were compared using one-way ANOVA for repeated measurement data to account for potential correlations among readings from the same rats.

### Statistical tests

The significance of differences in ONL thickness and GFAP labelling associated with conditioning were assessed using ANOVA, followed by a Tukey test. The Tukey test was used for all pairwise comparisons of the mean values. Results are expressed as the mean ± SE. *p<0.05* was considered significant.

## Results

### Single and combined conditioning: rate of photoreceptor death

**Figure 1:**
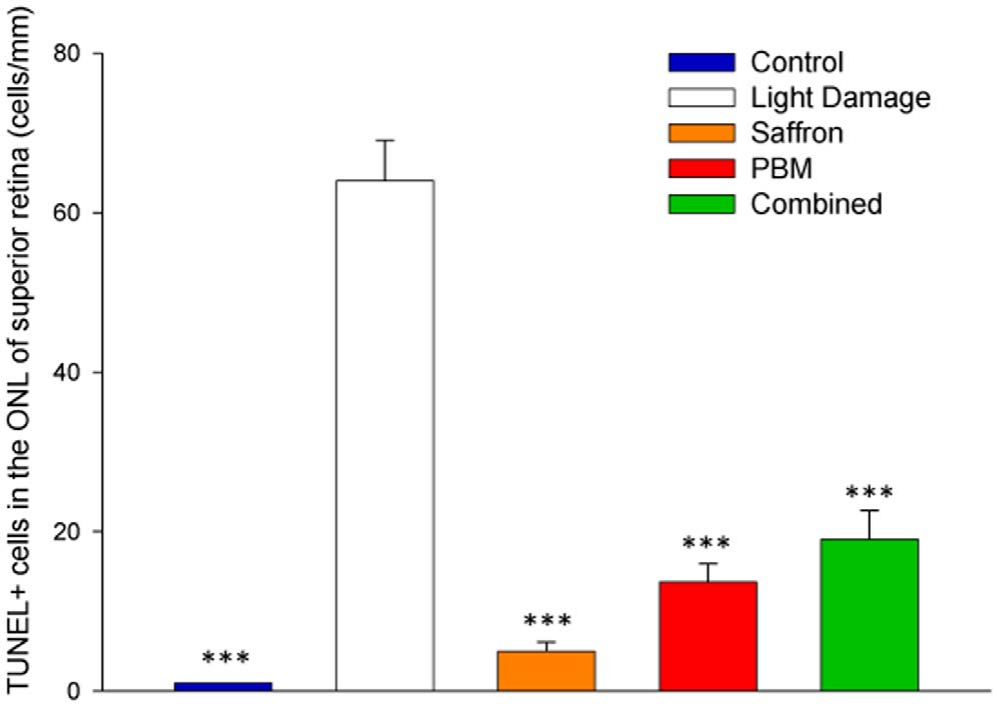
Impact of single and combined neuroprotectants on TUNEL-positive cells in the ONL of superior retina one week after light exposure. We tested whether the number of TUNEL+ cells were significantly different among 5 experimental groups. In all three treated groups, the number of TUNEL+ profiles was significantly smaller than in the light damage (LD) group. The histogram bars show mean numbers of TUNEL+ cells/mm ONL, for each experimental group; the error bars show standard error of the means. Statistical significance indicator: ^(^***^)^ p<0.001.

In control retina (dim-reared, not exposed to bright light, unconditioned by saffron or PBM) the rate of photoreceptor death, assessed by the frequency of TUNEL+ cells in the ONL (Figure 1, blue bar) was low (Figure 1). Exposure to damaging light (white bar) increased the count of TUNEL+ cells, most prominently in superior retina [13] (for reasons discussed in Section 3.2.2). When the retina was preconditioned with 7 d PBM, 10 d saffron or with both saffron and PBM, the TUNEL count in superior retina was reduced (orange, red, green bars). Because, in the current experiments, retinas were examined 1 w after the bright light exposure that induced cell death, the numbers of TUNEL+ profiles were lower than in earlier studies [4], in which the retina was examined immediately after damaging light exposure. For all three treatments a reduction in cell death from the unconditioned light damage level was evident and significant, but greater reduction was not achieved with PBM and saffron given simultaneously.

### Single and combined conditioning: photoreceptor survival

**Figure 2:**
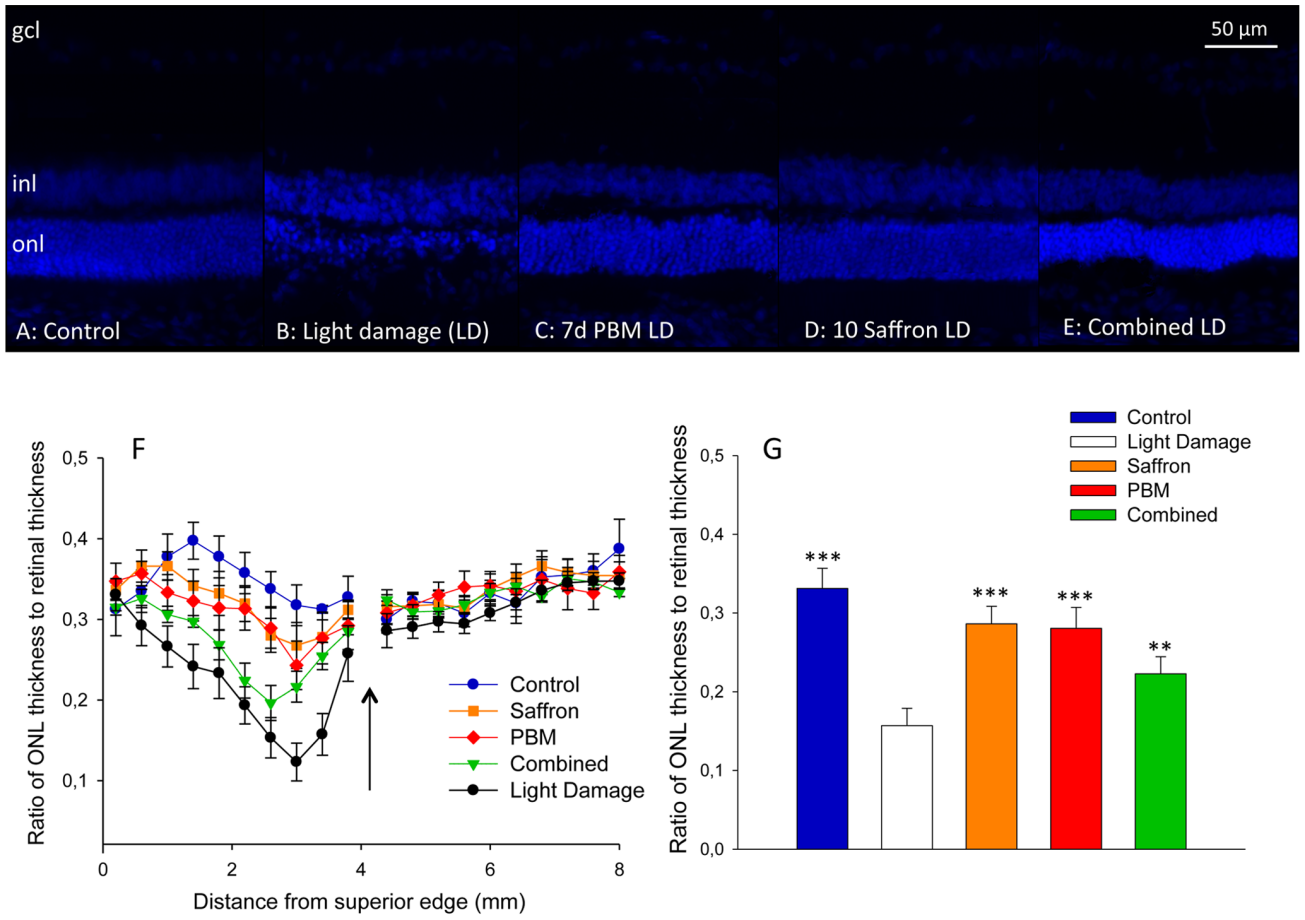
Impact of single and combined neuroprotectants on the thickness of the ONL in superior retina. A–E: Representative bisbenzimide labelling in control (A), light damage (B), 7 days PBM (C), 10 days saffron (D) and combined treatment (E) groups, 1 w after light damage. Images are taken one millimeter dorsal from optic disc. F: The ratio of ONL thickness to retinal thickness from the superior to the inferior edge. The arrow shows the position of the optic disc. Different symbols represent different experimental groups. For each group, each point shows the mean of the group; the error bars show standard errors of the mean. G: The ratio of ONL thickness to retinal thickness, in the hot spot area (the area of greatest light-induced damage), 1 mm superior to the optic disc. For each group, each point shows the mean of the group; the error bars show standard errors of the mean. Statistical significance indicators: ^(^***^)^ p<0.001; ^(^**^)^ p<0.01, for the difference of each group from the light damage group value.

Figure 2 shows representative images of superior mid-peripheral retina, in a control retina (A), and in retinas from the four experimental groups (B-E). The ONL is 50-80 µm thick in the control retina (A), and is sharply reduced in thickness by exposure to bright light for 24 h (B). Preconditioning with PBM (C) or saffron (D) was protective, limiting the thinning on the ONL; this confirms prior reports [1], [4], [5], [14]. Conditioning with both PBM and saffron (E) was also protective, but did not improve survival, compared to saffron or PBM conditioning separately.

The result is shown quantitatively in Figures 2F, G, which show ONL thickness as a function of distance between the superior and inferior edges of the retina. ONL thinning induced by LD is most marked in the superior retina (as reported in [15], [16]); the thinning is reduced by conditioning. Figure 2G shows the mean ONL thickness averaged across superior retina, in the five experimental groups. Comparisons were made using the four measures of ONL thickness available between 2.2 mm and 3.4 mm from the superior edge, thus excluding all inferior retina, and the more peripheral regions of superior retina. The difference between the unconditioned and PBM conditioned light damage groups was highly significant (*p<0.0001*), as were the differences between the saffron-conditioned and unconditioned groups (*p<0.0001*), and between the combined-conditioned and unconditioned groups (*p<0.0004*). The thinning of the ONL in the combined conditioned group, relative to the single conditioned groups, was significant for both saffron (*p<0.0001*) and PBM (*p<0.001*). The difference in thickness between PBM-and saffron-conditioned groups was not significant in these data (*p = 0.0626*; Figure 2G). In conclusion, combining saffron and PBM was not additive; indeed the thinning of the ONL was greater in the combined group than in the PBM or saffron groups.

### Single and combined conditioning: impact on GFAP expression

**Figure 3:**
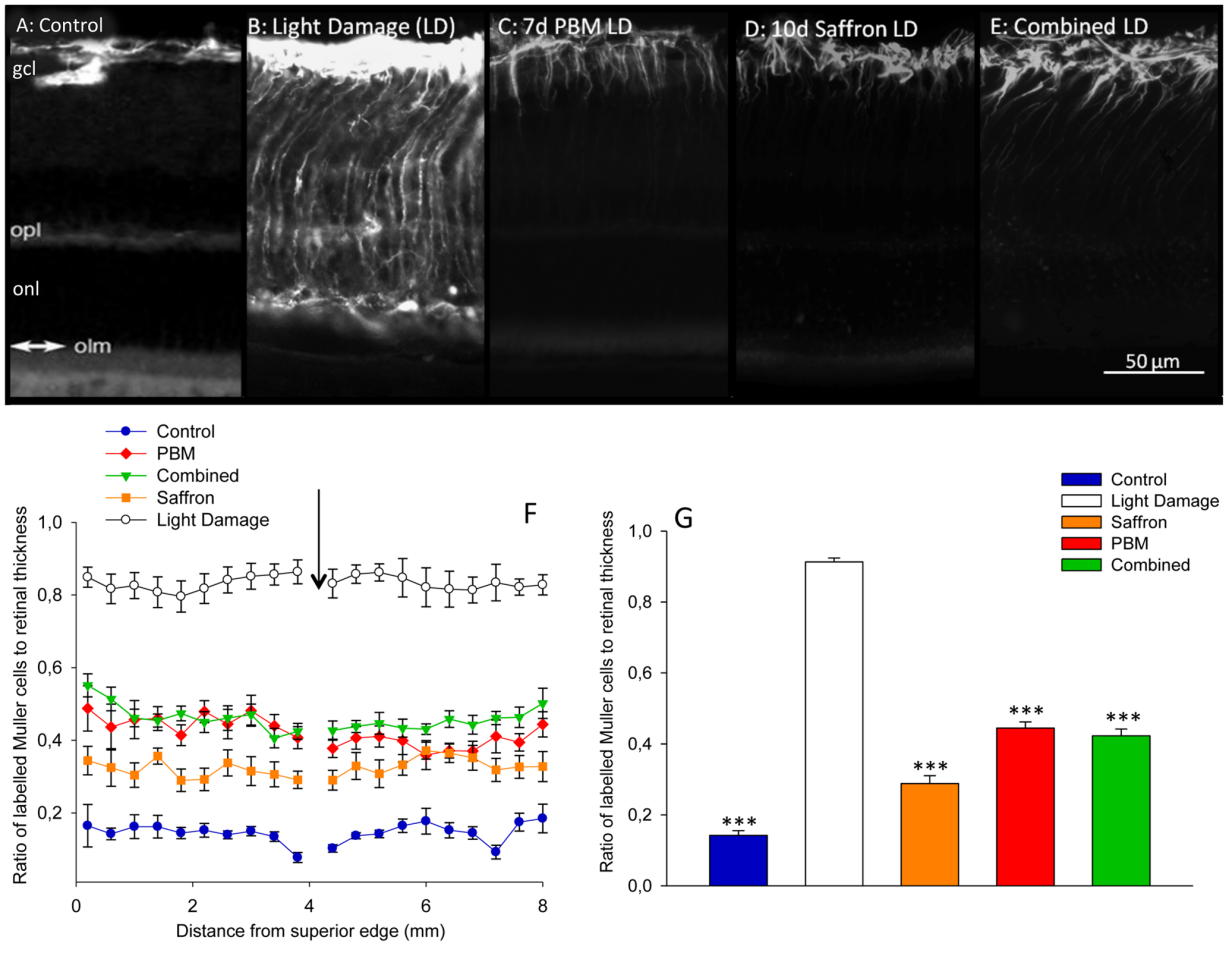
Impact of single and combined neuroprotectants on GFAP labelling of Müller cells. A–E: Representative GFAP labelling in control (A), light damage (B), 7 d PBM (C), 10 d saffron (D) and combined treatment (E) groups, 1 w after light damage. Light damage induced the up-regulation of GFAP in the radially oriented Müller cells; the protein is visible along the full length of the Müller cells, from the ILM to the OLM. F: Length of the Müller processes expressing GFAP as a function of distance from superior edge in the five experimental groups. The arrow shows the position of the optic disc. Each point shows the mean labelled length; error bars show standard deviations of the mean. G: Mean length of Müller processes expressing GFAP, averaged across superior retina, in: Control (A), Light Damage (B), 7 days PBM (C), 10 days saffron (D) and combined (E) groups. In all treated groups, the length of Müller cells expressing GFAP was less than in the light damage group. The error bars show standard errors of the mean. Statistical significance indicator: ^(^***^)^ p<0.001.

Figure 3 shows GFAP expression in the retina, in the five experimental groups. Without light damage or conditioning, GFAP expression is confined to the astrocytes at the inner surface of the retina (Figure 3A). Light damage induced the upregulation of GFAP in the radially oriented Müller cells; the protein was visible along the full length of the Müller cell (Figure 3B). The length of the Müller cell that expressed GFAP was shortened by 7d conditioning with PBM and by 10d conditioning with saffron, confirming previous study [5] (Figures 3C, D). The length of Müller cells labelled by combined conditioning is shown in Figure 3E.

The result is shown quantitatively in Figures 3F, G. The length of Müller cells labelled was consistent along sections of the retina, extending from the superior to the inferior edge. The difference in the length of Müller cell that was GFAP+ was highly significant between unconditioned and PBM conditioned light damaged groups (*p<0.0001*), between unconditioned and saffron conditioned groups (*p<0.0001*) and between the unconditioned and combined conditioned groups (*p<0.0001*; Figure 3G). That is, the unpregulation in GFAP expression caused in Müller cells by light damage was reduced by saffron, by PBM and by combined conditioning. As with the ONL, combined conditioning did not provide greater reduction in GFAP labelling than saffron single conditioning or PBM single conditioning. The biggest difference between the ONL and GFAP measures was that combined conditioning reduced GFAP upregulation less than saffron conditioning, but not less than PBM conditioning; saffron-conditioning reduced GFAP labelling significantly more than did PBM-conditioning (*p<0.0001*; Figure 3G).

### Single and combined conditioning: retinal function

**Figure 4:**
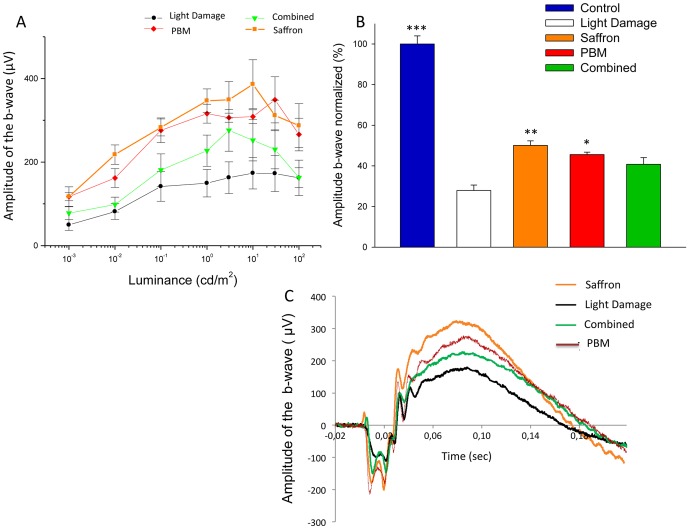
Impact of single and combined neuroprotectants on the ERG. A: ERG b-wave amplitude (µV) as a function of flash brightness (cd/m^2^) in four experimental groups. As for other data, each point represents the group mean and error bars show standard deviations of the mean. B: The amplitude of the b-wave normalized to control values in the 5 experimental groups. Histogram bars show means for each experimental group; error bars show standard deviations of the mean. The comparisons were made from data obtained at a fixed value of luminance (10 cd/m^2^) before saturation. Single treatment with saffron and PBM mitigated the b-wave reduction induced by light damage, and the differences between treated and untreated groups were significant. Combined treatment also mitigated the reduction of the b-wave, but the difference was not statistically significant. C: Representative traces for each group to a stimulus flash of 10 cd/m^2^ of luminance. The error bars show standard errors of the mean. Statistical significance indicators: ^(^***^)^ p<0.001; ^(^**^)^ p<0.01, ^(^*^)^ p<0.05.

Figure 4 shows the impact of PBM, saffron and combined conditioning on the preservation of retinal function. We recorded ERG responses as a function of increasing flash intensity (Figure 4A), from threshold to saturation. Figure 4C shows representative ERG traces obtained at a fixed luminance (10 cd/m^2^), less than saturation. Quantitative results for different treatments are summarized in Figure 4B as a percentage of control amplitude of the b-wave. The amplitude was strongly reduced after light damage (70% reduction). Pre-conditioning with 7 d PBM and 10 d saffron mitigated the reduction, to 40% and 50% respectively. Preconditioning with both PBM and saffron, given simultaneously did not increase the mitigation. We recorded responses as a function of increasing luminance from threshold to saturation. Comparison were made from data obtained at a fixed value of luminance (10 cd/m^2^), which was non-saturating.

The difference in the amplitude of the b-wave is significant between unconditioned and PBM conditioned light damaged group (*p<0.05*) and between unconditioned and saffron conditioned light damaged group (*p<0.01*; Figure 4B). The difference between unconditioned and combined conditioning is not significant (*p = 0.244*; Figure 4B). In agreement with morphologic data, these data demonstrate that both treatments are effective in preserving retinal function when separately applied but their protective efficacy is lower, and in this case not significant, when simultaneously applied.

## Discussion

The present observation, that neuroprotective effects of saffron and PBM are not additive, does not support our working hypothesis, formulated on the basis of results obtained in a microarray study [9], where we observed a limited overlap of gene regulation patterns during saffron- and PBM-induced neuroprotection. We hypothesized from that data that saffron and PBM would activate separate but complementary, and therefore potentially additive, protective pathways. The lack of any additive effect suggests that saffron and PBM compete to activate the same neuroprotective pathway or pathways. The dose-effect data in a previous study [5] suggest that the mechanism involved is activated progressively, over 5–10d, but further work is required for it to be identified.

The experimental stress we used (bright continuous light exposure) induces oxidative stress, to which neurons are vulnerable. Oxidative stress can also contribute to tissue damage indirectly, by activating pathways that induce the expression of stress sensitive genes, and by glia-mediated inflammation that causes secondary neuronal damage [17]. The neuroprotective activity of saffron probably is not limited to the trapping of free radicals, as an antioxidant, but likely involves regulation of genes which control the release of pro-inflammatory cytokines by glial cells. While acting as neuroprotectants, both saffron and PBM downregulate chemokine gene expression [9].

### Do PBM and saffron interfere with each other's actions?

In both the ONL and GFAP data, combined treatment gave a poorer outcome than in single saffron conditioning; the differences were statistically significant. This suggests a limited degree of interference between the two forms of conditioning. Confirmation of this interference and more knowledge of the underlying mechanisms seem necessary for progress towards the successful combination of neuroprotectants. The suggestion of interference gives weight to the main outcome of this study, that the protective effects of PBM and saffron are not additive; greater protection is not gained when both forms of conditioning are applied.

More positively, our results show that combination of saffron and PBM preconditioning does not introduce major side effects or induce a major reduction in neuroprotection. The general idea of combining different treatments to reach a better results remains valid, but needs further investigation to form a basis in animal models for human trials of combinations of protectants.
